# Continued Decline of Malaria in The Gambia with Implications for Elimination

**DOI:** 10.1371/journal.pone.0012242

**Published:** 2010-08-18

**Authors:** Serign J. Ceesay, Climent Casals-Pascual, Davis C. Nwakanma, Michael Walther, Natalia Gomez-Escobar, Anthony J. C. Fulford, Ebako N. Takem, Sarah Nogaro, Kalifa A. Bojang, Tumani Corrah, Momodou Cherno Jaye, Makie Abdoulie Taal, Aja Adam Jagne Sonko, David J. Conway

**Affiliations:** 1 Medical Research Council Laboratories, Fajara, Banjul, The Gambia; 2 Wellcome Trust Centre for Human Genetics, University of Oxford, Oxford, United Kingdom; 3 Department of Infectious & Tropical Diseases, London School of Hygiene & Tropical Medicine, London, United Kingdom; 4 National Health Laboratory Services, Royal Victoria Teaching Hospital, Banjul, The Gambia; 5 National Public Health Laboratories, Kotu, Banjul, The Gambia; 6 National Malaria Control Programme, Banjul, The Gambia; Université Pierre et Marie Curie, France

## Abstract

**Background:**

A substantial decline in malaria was reported to have occurred over several years until 2007 in the western part of The Gambia, encouraging consideration of future elimination in this previously highly endemic region. Scale up of interventions has since increased with support from the Global Fund and other donors.

**Methodology/Principal Findings:**

We continued to examine laboratory records at four health facilities previously studied and investigated six additional facilities for a 7 year period, adding data from 243,707 slide examinations, to determine trends throughout the country until the end of 2009. We actively detected infections in a community cohort of 800 children living in rural villages throughout the 2008 malaria season, and assayed serological changes in another rural population between 2006 and 2009. Proportions of malaria positive slides declined significantly at all of the 10 health facilities between 2003 (annual mean across all sites, 38.7%) and 2009 (annual mean, 7.9%). Statistical modelling of trends confirmed significant seasonality and decline over time at each facility. Slide positivity was lowest in 2009 at all sites, except two where lowest levels were observed in 2006. Mapping households of cases presenting at the latter sites in 2007–2009 indicated that these were not restricted to a few residual foci. Only 2.8% (22/800) of a rural cohort of children had a malaria episode in the 2008 season, and there was substantial serological decline between 2006 and 2009 in a separate rural area.

**Conclusions:**

Malaria has continued to decline in The Gambia, as indicated by a downward trend in slide positivity at health facilities, and unprecedented low incidence and seroprevalence in community surveys. We recommend intensification of control interventions for several years to further reduce incidence, prior to considering an elimination programme.

## Introduction

Malaria elimination is being considered as a decisive long-term solution to the intolerable burden of this disease on a large proportion of the world's poor communities. Although the potential for eliminating malaria from major endemic regions of the African continent is not yet clear [1], there has been increasingly effective control in countries on the fringe of malaria distribution, including South Africa [2], [3] and neighbouring parts of Swaziland and Mozambique [3], as well as Eritrea [4]. The possibility of local elimination from islands is also apparent, as intensified malaria control has reduced incidence to very low levels in Zanzibar [5], Sao Tome and Principe [6], and Bioko in Equatorial Guinea [7]. Although there are few datasets from previously highly endemic countries, we reported a major decline in numbers and proportions of positive slide examinations, admissions and deaths due to malaria in The Gambia over several years until the end of 2007 [8]. There have also been major reductions in malaria at the Kenyan coast [9] and in some communities nearby in Tanzania [10], but analysis of hospital records from different parts of Kenya has shown substantial variation in trends, indicating that declines in malaria are not universal [11].

It is important to critically evaluate prospects for malaria elimination [12], [13], recognising that there are situations in which this goal is not achievable in the foreseeable future [14]. A country or regional partnership experiencing success in malaria control has to consider whether to scale up to a phase of ‘pre-elimination’ [12], [13], with enhanced control and surveillance aiming to reduce incidence to a minimal level ahead of attempting elimination, and epidemiological milestones are needed to guide this. Although there are now more refined epidemiological models compared to those that encouraged the Global Malaria Eradication Programme in 1955–1969 [15], [16], practical surveillance and data on malaria in many countries are poorer today than they were 50 years ago in those that undertook malaria elimination attempts [17]. Analysis of trends in imported malaria in the United Kingdom has indicated a slight decrease in risk of malaria among people arriving from West Africa over the last 20 years [18], but there are few published data directly from countries in this large region of endemicity. Apart from The Gambia, limited data suggest that malaria has declined in Guinea Bissau [19], [20] but not in Burkina Faso [21].

Surveillance of malaria trends is now widely recognised as a long-overlooked priority for public health. Although modelling efforts have improved ability to extrapolate estimates from available data [22], a general lack of specificity in diagnosis and poor recording of data make it difficult to reliably detect changes in the malaria burden over extensive and economically-disadvantaged endemic populations, Numbers of presumptive malaria cases are the most commonly available data, but slide-confirmed cases should provide more specific indices. Higher quality data tend to be available from a limited number of sites that are well resourced, often linked with long-term medical research programmes, and may be those at which a reduction in malaria has been most marked due to a bias in access to prevention and treatment.

Our previous description of the substantial decline in malaria in The Gambia up until the end of 2007 [8] was supplemented by cross-sectional surveys at the beginning of 2008 [20]. These studies were in response to a changing situation and thus could not be comprehensive, but they stimulated early discussions on the possibility of local elimination of malaria. Here we present analysis of laboratory slide examination records from 10 health facilities in all five administrative regions of the country for the seven year period up until the end of December 2009, together with community surveys conducted in the past two years on infection incidence and serology in communities that were previously highly endemic.

## Methods

### Ethics Statement

The overall study and its components were reviewed and approved by the MRC Gambia Scientific Co-ordinating Committee, and the Gambia Government and MRC Joint Ethics Committee.

### Study sites and populations

**Figure 1:**
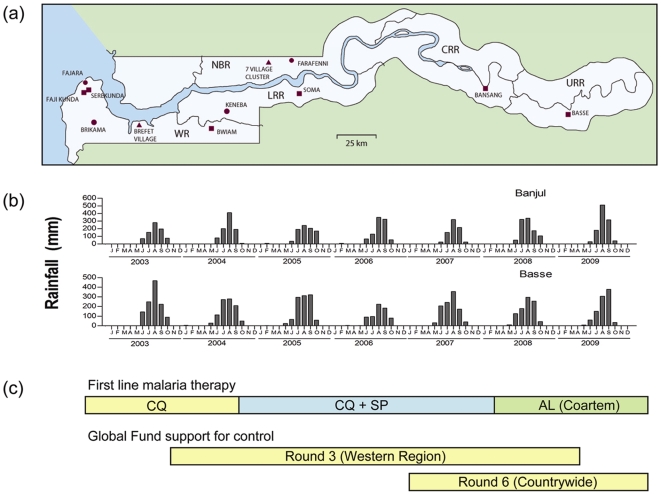
Location of health facilities and populations surveyed, rainfall patterns and Global Fund support for malaria control from 2003 to 2009 in The Gambia. (a) Sites of 10 health facilities at which laboratory slide data were analysed (circular symbols indicate 4 sites previously studied until the end of 2007, square symbols indicate 6 sites not previously studied); sites of the Farafenni village cluster and Brefet village are marked with triangular symbols. (b) Monthly rainfall for two sites at opposite ends of the country (Banjul at the coast in the west and Basse in the east) over the 7 year period. (c) Changes in first-line antimalarial therapy and Global Fund support for malaria control over the period. First-line therapy: CQ, Chloroquine; SP, Sulphadoxine-Pyrimethamine; AL, Artemether-Lumefantrine. The Global Fund has supported Insecticide treated bednet (ITN) provision for children 5 years of age and pregnant women, and intermittent preventative treatment with SP during pregnancy (IPTp) under Round 3 from 2004 onwards (Western Region) and Round 6 from 2007 onwards (countrywide), as well as the purchase of AL (Coartem) to allow free treatment with an effective artemisinin-based combination therapy from 2008 onwards. Coverage of interventions countrywide by the midpoint year (2006) reached 50% for ITN use by children under 5 years of age and 30% for receipt of at least 2 doses of IPTp [35].

This study was performed in The Gambia, a country on the coast of West Africa in which the epidemiology of malaria has been extensively described in studies over the past 60 years [8], [20], [23], [24]. Locations of the 10 health facilities from which we have surveyed the numbers and proportions of positive malaria blood slide examinations from January 2003 up to December 2009 are shown in Figure 1A. In the Western Region, we studied five facilities: Medical Research Council (MRC) hospital in Fajara; and the Government Major Health Centres in Serekunda, Fajikunda, and Brikama, and Sulayman Junkung Memorial Hospital in Bwiam. In the Lower River Region, we analysed laboratory haematology data from Soma Major Health Centre, and the MRC Clinic in Keneba. We also analysed haematology laboratory data from the Armed Forces Provisional Ruling Council (AFPRC) District Hospital in Farafenni in the North Bank Region, Bansang General Hospital in Central River Region, and Basse Major Health Centre in the Upper River Region. Malaria slide positivity is not differentiated into parasite species in these facilities, as *Plasmodium falciparum* is the species responsible for almost all malaria cases in The Gambia.

A community cohort study of malaria incidence was performed from August 2008 until January 2009 in a cluster of seven rural villages (Alkali Kunda, Jarjari, India, Yallal, Daru, Jumansareba and Conteh Kunda) situated 5–15 km west of Farafenni in the North Bank Region, within the Farafenni Demographic Surveillance System. For a comparison of population serological profiles over time, a cross-sectional survey was performed in Brefet village in the Western Region in December 2009 and samples assayed alongside those sampled in December 2006 [25] that were re-assayed here. All subjects or their parents or guardians gave written informed consent to participate in these studies.

Rainfall throughout The Gambia is highly seasonal every year, increasing progressively from June to August, and continuing heavily in September before diminishing in October, as shown by data from 2003–2009 from Banjul (at the coast in the west) and Basse (inland in the east) (Figure 1B). Malaria incidence follows this pattern, occurring predominantly during and immediately after the rainy season, with an annual peak typically in the month of October. Changes in first-line treatment for malaria and major milestones in support for malaria control at the country level over the 7 year period are indicated in Figure 1C.

### Data collection from health facilities

We examined laboratory records for the past 7 years from January 2003 to December 2009 from 10 health facilities. We counted individual blood slide microscopy records from the complete series of laboratory books of the MRC outpatient haematology laboratory in Fajara, the MRC clinical laboratory in Keneba, and facilities at Brikama and Farafenni which had established links with the MRC for laboratory quality control and training in slide microscopy (data for these four sites up until the end of 2007 were previously analysed as part of our earlier study) [8]. Data were also collected by counting individual blood slide microscopy records in laboratory books from 6 other facilities in the country (at Fajikunda, Serekunda, Bwiam, Soma, Bansang and Basse) that did not have continuous quality control and training throughout the period. Quality control procedures were introduced in 2007 for Fajikunda, Serekunda, and Bwiam, and in 2008 for Bansang, Basse and Soma, with support from the Global Fund and Irish Aid.

### Geographical mapping of malaria cases

From two of the health facilities (Fajara and Brikama) a subset of 285 malaria patients with >5000 *P. falciparum* parasites µl^−1^ living in the Kombo coastal districts had participated in a study of clinical malaria in 2007 – 2009, and a trained fieldworker located the residential compound of each and entered its geographical location with a GPS device (Garmin GPS 12XL). These subjects were part of a study that excluded a small number of residential areas immediately on the north and west coasts where malaria incidence was known to be extremely low (Banjul, Cape Point, Bakau, Fajara, Bijilo, Kololi, Kotu). These data were transferred to a computer using DNR Garmin software (Minnesota Department of Natural Resources) and saved using projected coordinate system Universal Transverse Mercator (UTM) and World Geodetic System (WGS) 84 map datum. A map (produced by the Japanese International Corporation Agency) with the same format was used to overlay our spatial data with Arc GIS version 9.3 software (ESRI, Redlands, CA, USA).

### Malaria serology

Two cross-sectional serological surveys of the population of Brefet village in Western Region were performed, in December 2006 (N = 211) and December 2009 (N = 98), each at the end of the annual transmission season when the proportion of the population exposed and antibody positive was expected to be highest. Plasma IgG levels binding to a recombinant protein representing the 19-kilodalton C-terminal part of merozoite surface protein-1 (MSP-1_19_ Wellcome allele fused to glutathione S transferase) were measured by indirect ELISA, using an assay protocol previously described [20], [26]. Samples from 2006 had been obtained for a previous study [25], and were re-tested alongside the samples from 2009 under identical assay conditions at a single dilution (1/1000). ELISA reactivities were considered positive when the OD value was above the upper limit of a 99% CI interval defined for anti-MSP-1_19_ measurements in plasma from 20 malaria naïve tourists tested simultaneously within the same assay.

### Active case detection of malaria

A cohort of 800 children (408 females and 392 males) aged 1 to 15 years was recruited from the cluster of 7 villages near Farafenni, and studied throughout the annual malaria season from August 2008 to January 2009. Baseline malaria screening was conducted in each of the study villages over a three week period from 4^th^ to 22^nd^ August 2008, axillary temperature and finger prick blood sample for malaria microscopy were taken, and study nurses placed in the study villages to actively follow up the health status of the cohort for a period of 22 weeks from 25^th^ August 2008 to 24^th^ January 2009. Each study participant was visited at home weekly and examined by a nurse, and they and their guardians were instructed to consult the study nurses at any time between scheduled visits if they felt unwell. If clinical symptoms suggestive of malaria were detected at any time (including axillary temperature ≥37.5°C or a history of fever within the previous 48 hours) a rapid diagnostic test (RDT, Optimal, Diamed AG, Cressier, Switzerland) was performed and an appropriate course of artemether and lumefantrine (Coartem) therapy administered if positive. In such cases, a thick blood smear was also collected for subsequent confirmation of the diagnosis. An incident case of malaria was defined as any degree of parasiatemia detected in a thick smear in a study subject with fever or history of fever within the previous 48 hours without any other obvious cause for the fever.

### Data processing and statistical analysis

Laboratory data for each month at each health facility were transcribed into formatted tables, following which data were double entered into an MS Access database. A clean database was generated and converted using Stat Transfer (v8) for analyses using Stata 9 and Stata 11 software (Stata Corporation, Texas, USA). Least squares multiple regression of the logarithm of the number of positive malaria slides per month was used to investigate the seasonality and longer-term trends with time at each site. Seasonality was captured by fitting the first two pairs of Fourier series terms (by including sin*θ*, cos*θ*, sin2*θ* and cos2*θ* as covariates in the regression model, where *θ* is the angle representing the position in the annual cycle corresponding to the middle of the month), as previously applied to other seasonal health indices [27]. Further Fourier terms were found to have no significant effect, and trends were fitted either as a linear term or a linear term plus categorical term stratified by year, with significance evaluated using the Wald test. The average percentage decline in malaria per year and its 95% confidence interval for each site were estimated from the linear trend term and its standard error. Partial R^2^ correlation values were calculated to provide a measure of the size of the more complex effects of seasonality and year-to-year fluctuations over and above the linear trend.

## Results

From all of the 10 health facilities investigated (Figure 1), we were able to count individual laboratory records of 356,282 malaria slide examinations for the 7 year period from January 2003 to December 2009, with no more than one month missing data from any site except Bwiam Hospital (this facility opened in January 2004). Of these, 112,575 examinations had been conducted in four of the sites for the period 2003–2007 and analysed in our previous report [8], while 243,707 are unreported data from these sites over the past two years and from the other six sites over the whole period. Overall crude proportions of slides positive were reduced approximately 4-fold, from 32.8% (19,284/58,851) in 2003 to 8.5% (5145/60,669) in 2009, and the mean positivity across individual sites was reduced from 38.9% in 2003 to 7.2% in 2009.

**Figure 2:**
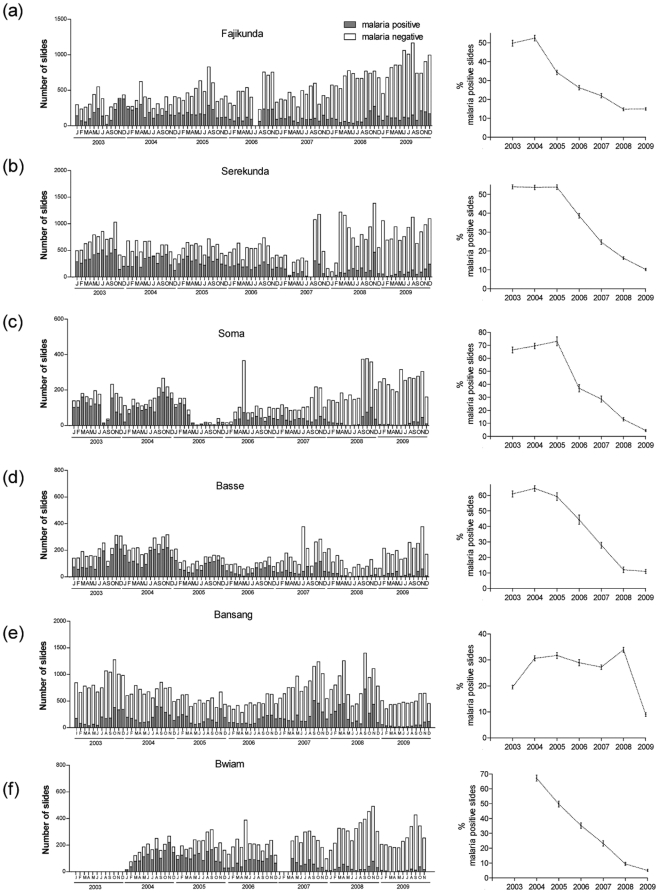
Malaria trends during 2003–2009 at 6 newly-surveyed health facilities in The Gambia. Monthly numbers (left panel) and annual proportions (right panel) of malaria positive slides from January 2003 to December 2009 at (a) Fajikunda Health Centre (b) Serekunda Health Centre, (c) Soma Health Centre, (d) Basse Hospital, (e) Bansang Hospital, and (f) Bwiam Hospital.

Each of the six newly studied health facilities showed highly significant declines in the numbers and proportions of malaria positive slides (Figure 2). Five of these had continuously significant annual declines over several years, from 2004 onwards (at Fajikunda and Bwiam) or 2005 onwards (at Serekunda, Soma, and Basse). At the remaining site (Bansang) there was a significant decline only after 2008. Comparing the last year (2009) with the first year in the data series (2004 for Bwiam, 2003 for other sites), proportions positive underwent reductions of approximately 14-fold in Soma (RR = 0.07, 95% CI 0.06–0.08) and Bwiam (RR = 0.07, 95% CI 0.06–0.09), approximately 5-fold in Basse (RR = 0.18, 95% CI 0.16–0.20) and Serekunda (RR = 0.19, 95% CI 0.18–0.20), approximately 3-fold in Fajikunda (RR = 0.30, 95% CI 0.29–0.32) and 2-fold in Bansang (RR = 0.46, 95% CI 0.42–0.50).

**Figure 3:**
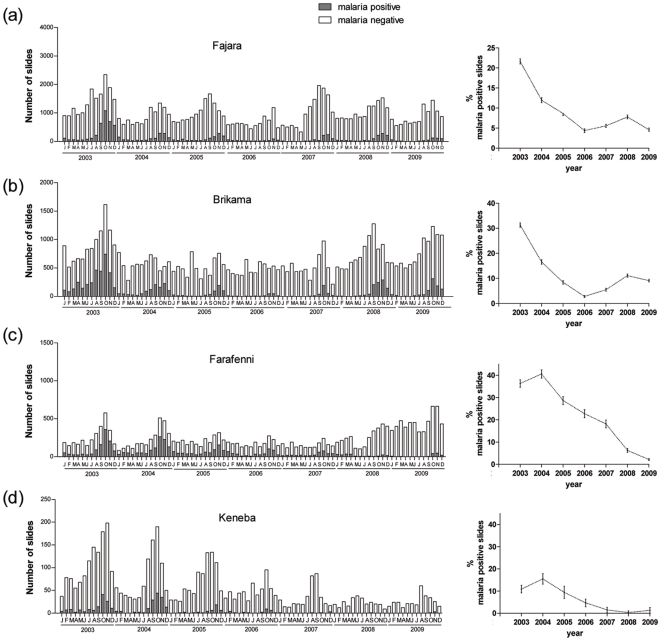
Malaria trends during 2003–2009 at 4 health facilities in The Gambia that had been previously surveyed until 2007. Monthly numbers (left panel) and annual proportions (right panel) of malaria positive slides from January 2003 to December 2009 at (a) Fajara MRC outpatient clinic, (b) Brikama Health Centre, (c) Farafenni AFPRC Hospital, and (d) Keneba MRC clinic.

All four sites for which data until 2007 had been previously reported [8] and that had laboratory quality control throughout the period of data recording also showed significant declines in the numbers and proportions of positive slides (Figure 3). Comparing 2009 with 2003, proportions positive underwent reductions of approximately 17-fold in Farafenni (RR = 0.06, 95% CI 0.05–0.07), 8-fold in Keneba (RR = 0.12, 95% CI 0.05–0.32), 5-fold in Fajara (RR = 0.21, 95% CI 0.19–0.23), and 3-fold in Brikama (RR = 0.29, 95% CI 0.27–0.31). The expected strong seasonality of malaria is highly evident at these sites, with few malaria positive slides during the dry season, contrasting with maximum numbers during and immediately following the annual rains. This seasonal pattern was also apparent at the other sites, although less marked due to a background of positive slides recorded during the dry seasons. Multiple regression analysis showed significant seasonality in numbers of positive slides at each of the ten sites, although this was stronger for those that had laboratory quality control throughout the period (Table 1, lower four sites). This analysis also confirmed year to year variation in numbers of slides positive. Fitting a linear model indicated average annual declines at each site of between 7.7% and 37.3%, although this simple model fitted poorly with data from some sites and other unknown causes accounted for between 4.6% and 40.4% of the overall year to year variation at each site (Table 1 and Figure S1).

**Table 1:** Modelling of seasonality and average annual decline in numbers of malaria positive slides at each of the 10 health facilities studied in The Gambia

	*Seasonality*			*Annual linear trend*	*Annual non-linear fluctuation*		
	F value	P value	R^2^ (%)	% decline (95% CI)	F value	P value	R^2^ (%)
Fajikunda	3.6	0.0098	15.0	7.7 (0.4–14.6)	2.1	0.0623	12.2
Serekunda	2.4	0.0558	7.5	27.1 (19.8–33.7)	2.6	0.0228	11.0
Soma	3.1	0.0204	9.4	30.7 (22.6–38.0)	3.2	0.0072	12.5
Basse	6.5	0.0002	12.8	33.6 (28.1–38.6)	6.7	<0.0001	13.8
Bansang	7.9	<0.0001	27.1	11.3 (4.1–17.9)	18.2	<0.0001	40.4
Bwiam	5.9	0.0004	15.0	37.3 (30.6–43.3)	3.1	0.0147	8.4
Fajara	43.0	<0.0001	58.8	26.6 (20.5–32.2)	8.1	<0.0001	10.8
Brikama	23.2	<0.0001	48.3	28.4 (19.5–36.3)	20.3	<0.0001	25.5
Farafenni	20.6	<0.0001	29.6	36.6 (31.4–41.3)	4.6	0.0005	7.7
Keneba	18.8	<0.0001	29.1	31.1 (26.4–35.6)	2.1	0.0645	4.5

**Figure 4:**
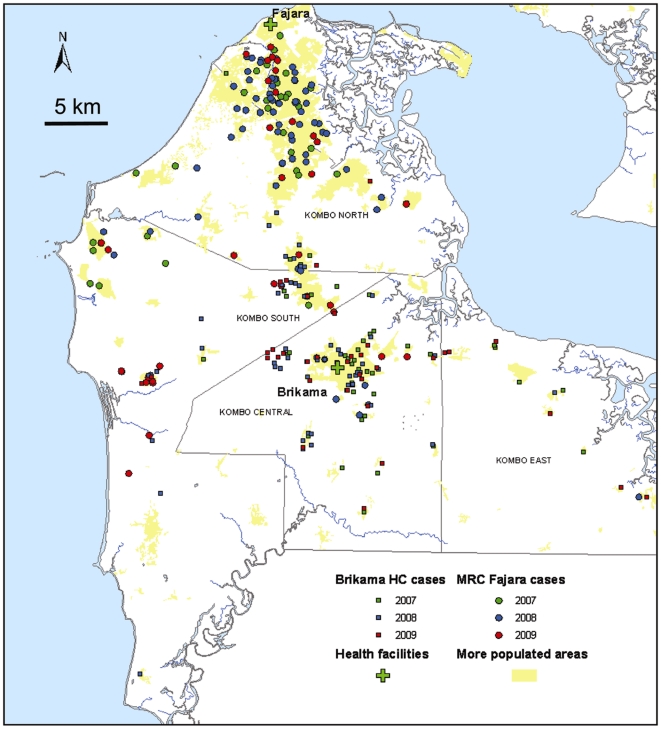
Spatial distribution of cases at two health facilities in the coastal area. Map of household locations of 285 malaria cases sampled from those presenting to the Fajara MRC clinic and Brikama Health Centre in three conseculive malaria seasons 2007 to 2009. Cases presenting to Fajara are represented as circular symbols, and those presenting to Brikama are represented with square symbols.The locations of these two health facilities are shown (green crosses), along with the most densely populated areas (shaded in yellow).

Two of the sites (Fajara and Brikama, both in the coastal Western Region) each showed continuous annual declines in malaria slide positivity down to <5% by 2006, after which proportions increased in the following two years before declining again, and we suspected that a few residual transmission foci (‘hotspots’) may have been responsible for this recent discontinuous trend. Therefore we analysed the geographical distribution of households of a sample of 285 confirmed malaria cases presenting to these facilities. This showed spatially broad distributions of these cases in each of the last three years, covering diverse urban and rural areas (Figure 4), with no strong evidence of aggregation other than that expected from crudely known population density combined with proximity to the health facilities.

Community-based studies were performed to identify infection trends in two different rural populations. In the cohort of 800 children aged 1–15 years in 7 villages west of Farafenni under active and passive case detection from August 2008 to January 2009, a total of 223 febrile episodes were recorded, of which 24 (11%) were associated with malaria parasites. Such episodes occurred in 22 (2.8%) of the children in the cohort overall, with two children having two episodes each. There was no significant difference in risk of malaria with age, 13 episodes occurring among 361 children aged 5 years and under, and 11 episodes among 439 children aged 6 years and above.

**Figure 5:**
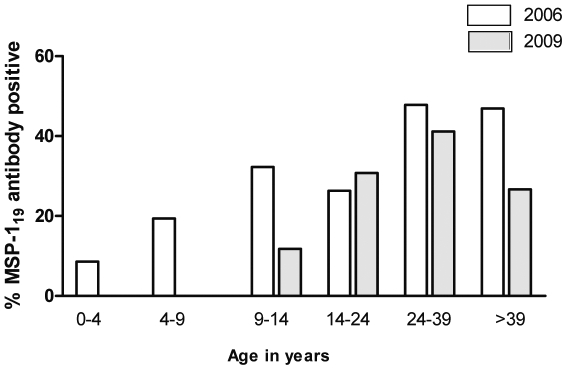
Serological surveys of malaria in Brefet village at the end of annual malaria seasons, December 2006 (n = 211) and December 2009 (n = 98). Proportions with detectable serum IgG to the MSP-1_19_ antigen are plotted by age group (numbers in 2006 and 2009 respectively sampled from each age group: 0–4 years, n = 35 and 17; 5–9 years, n = 31 and 19; 10–14 years, n = 31 and 17; 15–24 years, n = 19 and 13; 25–39 years, n = 46 and 17; >39 years, 49 and 15).

In Brefet village, close to the River Gambia in the Western Region, sero-prevalence of anti-MSP-1_19_ IgG antibodies was compared in plasma samples collected at the end of the transmission season (December) in 2006 and 2009. The proportion of MSP-1_19_ sero-positives was lower in 2009 than 2006 in all age groups (Figure 5). The most substantial difference between the years was apparent in children, with 19/97 (20%) of those 0–14 years of age being seropositive in 2006 compared to 2/53 (4%) in 2009 (p = 0.008). The absence of detectable antibodies in any child sampled under 10 years of age in 2009 indicates that transmission of malaria in this community has been minimal for several years.

## Discussion

The extensive assessment here of the profound country-wide decline of malaria in The Gambia allows informed discussions on the possibility of future elimination. Between 2003 and 2009, among patients presenting with symptoms for which a malaria slide examination was performed, proportions of positive slides declined significantly at all of the 10 health facilities studied in diverse urban and rural areas. By 2009, the slide positivity rate at each health facility was <15% (with a mean of 7.2%), and in half of these it was 5% or less. In a few sites there has been an increase in the numbers of slides examined over the past few years, due to an improvement in capacity, which could potentially magnify the recent decline in proportions positive at those sites. This illustrates the importance of looking at trends across diverse health facilities as we have done, rather than over-emphasising results from any one site, recognising that it is not possible to identify all site-specific factors that influence quantity and quality of slide reading.

Importantly, community-based surveys provide equally marked evidence that malaria in The Gambia has reached unprecedented low endemicity. In a cluster of rural villages near Farafenni, only 2.8% of a cohort of children experienced a clinical episode of malaria during the malaria season of 2008. This proportion was ∼10-fold lower than that seen in a cohort study in similar communities in the area only 4 years previously [28], and ∼20-fold lower than the seasonal incidence reported 20 years before that [24]. Cross-sectional serological surveys in another rural community at the end of the annual malaria seasons in 2006 and 2009 revealed substantial decline in the seropositive proportion of children under 15 years of age, from 20% to 4%. Such low malaria indices at health facilities and in communities have substantial implications for policies on health resource allocation and control or future elimination of the disease. As outlined in Figure 1, Global Fund support for targeted prevention in under 5 year old children and pregnant women began in the Western Region from the end of 2003 onwards and in the whole country from 2006 onwards, and there were changes in first line treatment practice from chloroquine alone to combination with sulphadoxine and pyrimethamine in late 2004, and to artemether-lumefantrane during 2008. Therefore, although the present study is not designed to infer causality, we note that the malaria decline has been quite coincident with improvement and scale up of malaria control, and its maintenance is likely to depend on this.

The potential for strategic orientation towards malaria elimination needs to be carefully undertaken at the country level where it might be practically implemented, as well as in regional and international partnerships that would be needed to guide and support such policy [29]. Recent guidelines from WHO recommended to consider entering a pre-elimination phase when the annual incidence of cases is reduced to <5 per 1000 population, with a proxy measure being the absence of any month of the year with slide positivity of >5% among suspected cases presenting to health facilities [12]. Data from the present study indicate that this milestone is being approached in The Gambia. However, there are heterogeneities among sites and we consider that enhanced control is needed to achieve consistent reduction of incidence by several-fold over at least five more years before a pre-elimination phase might be recommended.

It is salutary that an unusually large number of malaria cases was reported in tourists returning from The Gambia to several European countries at the end of 2008 [30]. Although such findings among groups of travellers mostly reflect variable awareness and use of prophylaxis, we note that most of these cases had stayed in the peri-urban coastal area served by health facilities at Fajara and Brikama, the only two sites at which we saw a rise in the number of cases in 2008. Local variation in malaria endemicity exists elsewhere in The Gambia [20], [31], and we consider it likely that there are discrete transmission foci or ‘hotspots’ remaining within the coastal area. The distributions of local cases presenting to the facilities we studied in Fajara and Brikama broadly reflected those of the catchment populations, and essentially indicates that some transmission persists in many of these coastal communities, although some clustering might be identified with more intensive sampling.

As The Gambia lies near the north-western limit of the endemic distribution of malaria in continental Africa [22], [32], together with Senegal and Mauritania to the north it would be a priority area for malaria elimination with a view to ‘shrinking the map’. However, universal reduction of transmission should first be achieved which requires increased capability and funding within governmental systems, and continued support from the Global Fund, UNICEF and many non-governmental organisations, to scale up existing available interventions and evaluate their effectiveness. There is also a continued need for creative research to identify which additional interventions are promising in particular settings [33], as well as those that are locally unsuitable [31]. Alongside this, it is vital to develop reliable surveillance systems based on effective and quality-controlled reporting of parasitologically confirmed cases of malaria. This priority has recently stimulated initiation of a project by the National Malaria Control Programme and the National Public Health Laboratories in The Gambia with support from development partners including Irish Aid, to strengthen routine surveillance at selected health facilities. Trans-national initiatives are also needed to share data and technical capabilities, and to incorporate partnerships with countries that have further to go in controlling malaria, particularly given the high levels of human migration that occur normally in this region [34].

## Supporting Information

Figure S1 Seasonality and temporal trends between January 2003 and December 2009 in numbers of malaria positive slides at each of 10 health facilities. (A) Plots show number of malaria-positive slides per month (blue columns) and seasonal trends specific to each site (yellow lines) as well as the average across all sites (red line) for comparison. (B) Average annual % declines (with 95% confidence intervals) at each of the 10 sites under a linear model. Statistical analyses are given in Table 1.
(0.43 MB PPT)
