# Supplementary figures and images for: Continued Decline of Malaria in The Gambia with Implications for Elimination
Source: PLoS One. 2010 Aug 18;5(8):e12242. doi: 10.1371/journal.pone.0012242 (PMC2923605; doi:10.1371/journal.pone.0012242)

## Slide 1
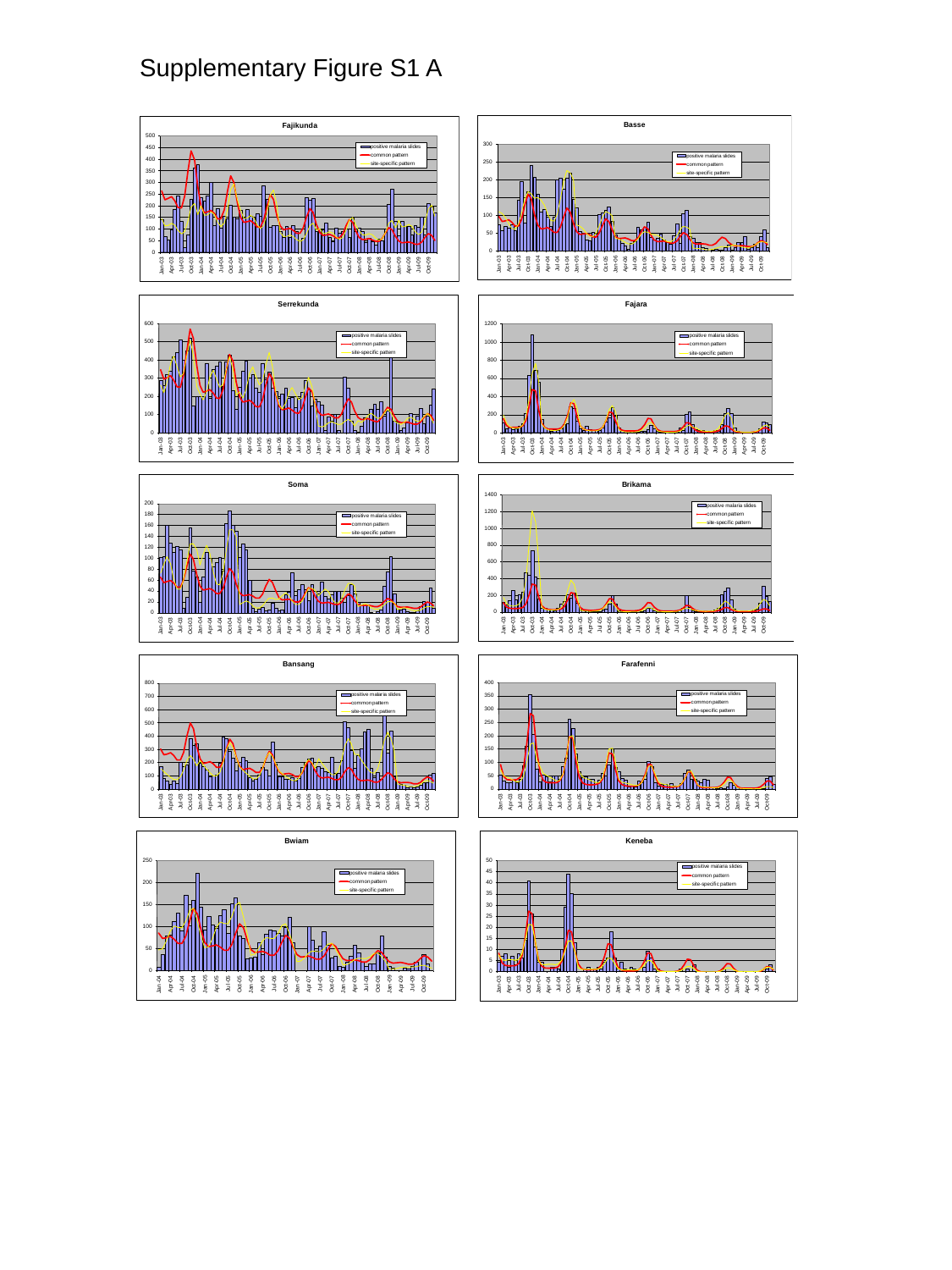

Supplementary Figure S1 A

## Slide 2
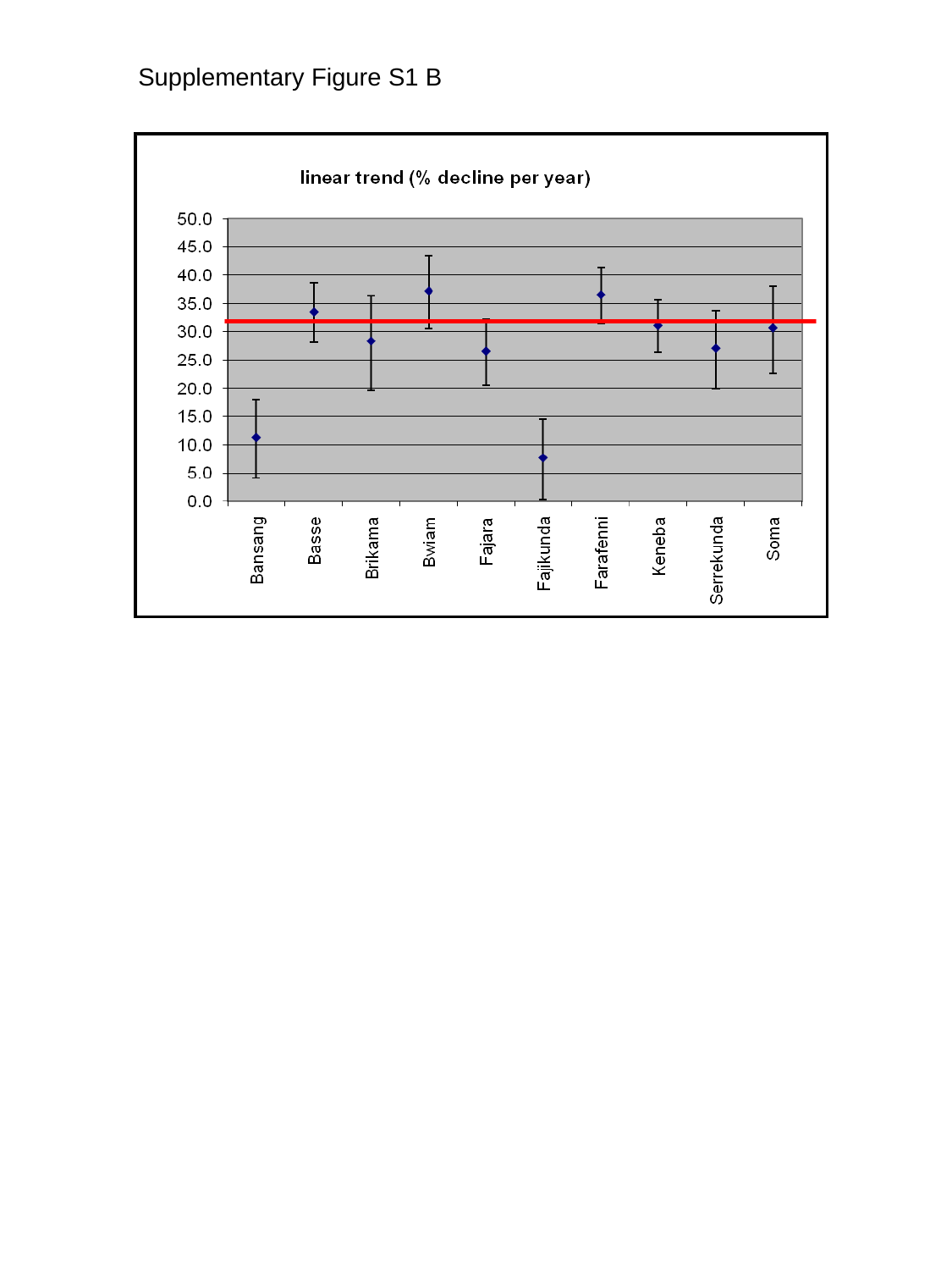

Supplementary Figure S1 B

Supplement: Figure S1 — Seasonality and temporal trends between January 2003 and December 2009 in numbers of malaria positive slides at each of 10 health facilities. (A) Plots show number of malaria-positive slides per month (blue columns) and seasonal trends specific to each site (yellow lines) as well as the average across all sites (red line) for comparison. (B) Average annual % declines (with 95% confidence intervals) at each of the 10 sites under a linear model. Statistical analyses are given in Table 1. (0.43 MB PPT) [file pone.0012242.s001.ppt]
